# Extracellular Vesicle-Associated miRNAs as a Biomarker for Lung Cancer in Liquid Biopsy

**DOI:** 10.3389/fmolb.2021.630718

**Published:** 2021-02-24

**Authors:** Xue He, Sujeong Park, Yan Chen, Heedoo Lee

**Affiliations:** ^1^ Department of Pulmonary and Critical Care Medicine, The Second Xiangya Hospital, Central South University, Changsha, China; ^2^ Department of Biology and Chemistry, Changwon National University, Changwon, Korea

**Keywords:** extracellular vesicles (EVs), lung cancer, microRNAs (miRNAs), bronchoalveolar lavage fluid (BALF), pleural lavage, blood

## Abstract

Extracellular vesicles are cell-derived membranous vesicles that are secreted into biofluids. Emerging evidence suggests that EVs play an essential role in the pathogenesis of many diseases by transferring proteins, genetic material, and small signaling molecules between cells. Among these molecules, microRNAs (miRNAs), a type of small noncoding RNA, are one of the most important signals and are involved in various biological processes. Lung cancer is one of the leading causes of cancer-related deaths worldwide. Early diagnosis of lung cancer may help to reduce mortality and increase the 5 years survival rate and thereby reduce the associated socioeconomic burden. In the past, EV-miRNAs have been recognized as biomarkers of several cancers to assist in diagnosis or prognosis. In this review, we discuss recent findings and clinical practice for EV-miRNAs of lung cancer in several biofluids, including blood, bronchoalveolar lavage fluid (BALF), and pleural lavage.

## Introduction

Extracellular vesicles (EVs) are a series of vesicles about 50 nm–1 μm in diameter that are delimited by a lipid bilayer. EVs are released from most cell types and can be detected in several biofluids, including blood, urine, sputum, and bronchoalveolar lavage fluid (BALF) (Xu et al., 2016). The membrane of EVs is similar to that of their “mother cells”, which also comprise a phospholipid bilayer (Raposo and Stoorvogel, 2013). Diverse materials are found within the EV membrane, including RNA, DNA, proteins, and other molecules (Raposo and Stoorvogel, 2013). It is believed that EVs, together with these materials, could act as a messenger between cells (Densmore et al., 2006; Tkach and Thery, 2016; Aiello et al., 2020). Emerging evidence shows that EVs play an important role in the pathogenesis of diseases (Kubo, 2018; Hill, 2019). Moreover, EVs have been recognized as a biomarker of these diseases and can help in diagnosis or prognosis (Becker et al., 2016; Jansen et al., 2017; Rahbarghazi et al., 2019).

MicroRNAs (miRNAs), which comprise 20–22 nucleotides, are a class of small noncoding RNAs. miRNAs have been shown to play important roles in various biological activities, such as cell proliferation, cell death, inflammation, tumorigenesis, and angiogenesis (O'Brien et al., 2018). Increasing evidence suggests that circulating or local miRNAs that are involved in cancer development can be detected and identified (Wang et al., 2018); they can be released into biofluids via the decomposition of dying cells or may be packed in EVs from live cells or together with RNA-binding proteins (Zhu and Fan, 2011). Importantly, it is now generally accepted that EVs provide protection, resulting in the enrichment of stable miRNAs whether in biofluids or extreme conditions (Fabris and Calin, 2016; Momen-Heravi et al., 2018). Furthermore, the isolation of EVs improves the sensitivity of miRNA detection from human biofluids (Gallo et al., 2012).

Lung cancer is the leading cause of cancer-related deaths worldwide with its morbidity still the highest among cancers (Malvezzi et al., 2017; Bray et al., 2018). About 80%–90% cases of lung cancer are non-small cell lung cancer (NSCLC), which includes squamous cell carcinoma (SCC), large cell carcinoma, and adenocarcinoma (Molina et al., 2008; Jemal et al., 2011), and the number of diagnosed cases of small cell lung cancer (SCLC) has been decreasing in many countries over the past 2 decades (Molina et al., 2008; Jemal et al., 2011). Although SCLC responds well to chemotherapy and radiation therapy, it may relapse and undergo rapid growth. The 5 years survival rate of NSCLC is about 15% (Molina et al., 2008) while for SCLC, it is lower than 7% (Byers and Rudin, 2015), which represents a huge threat to human health. Two-thirds of lung cancer patients are diagnosed with locally advanced or metastatic disease at the initial diagnosis (Molina et al., 2008). Different cancer types and stages are recognized followed by appropriate therapy (Fruh et al., 2013; Postmus et al., 2017; Planchard et al., 2018). For the stage I or II NSCLC patients, surgical resection is recommended. Surgical resection remains the most successful and beneficial therapy for NSCLC patients with stage I and II (Postmus et al., 2017). However, for the late stages (e.g., locally advanced or metastatic diseases), which accounts for the majority of diagnosed patients, chemotherapy, targeted therapy, and immunotherapy are the common and widely used treatments (Planchard et al., 2018). Based on molecular diagnostics of oncogenic driver, targeted therapies (e.g., epidermal growth factor receptor tyrosine kinase inhibitors) have been introduced for treating NSCLC patients (Modjtahedi and Essapen, 2009; Frezzetti et al., 2017; Guo et al., 2018). There are, however, still no approved drugs targeting SCLC oncogenes (Byers and Rudin, 2015). Overall survival (OS) of stage IV SCLC is less than 10 months (Fruh et al., 2013). Hence, the development of methods for early diagnosis, continuous monitoring and additional drugs is still urgently needed for lung cancer.

miRNAs and EVs have been shown to participate in the pathogenesis of cancers (Yang et al., 2011; Luga et al., 2012; Melo et al., 2014; Peinado et al., 2017). Moreover, it has been reported that miRNAs and EVs can be utilized in relation to diagnosis (Huang et al., 2014; Zhou et al., 2017), treatment response (Acharya et al., 2015; Dinh et al., 2016), or predicting prognosis (Wu et al., 2014; Shi et al., 2016). Aushev et al. analyzed 10 plasma samples of SCC patients and found that most species of miRNAs were enriched in exosomes, and not the exosome-free fraction (Aushev et al., 2013), indicating that EV-miRNAs may play an essential role in lung cancer. To date, there were already 18 clinical trials evaluating the role of exosomes in lung cancer, and four trials concerning EVs in lung cancer registered on clinicaltrials.gov. In this review, we discuss EV-miRNAs as a potential tool for liquid biopsy in various samples of lung cancer patients derived from serum, plasma, BALF, and pleural lavage.

## Blood EV-miRNAs in Lung Cancer

### Diagnostic Value

In 2009, Rabinowits et al. detected 12 specific miRNAs in both plasma epithelial-derived exosomes and tumor tissues (Rabinowits et al., 2009) (Table 1). These miRNAs had demonstrated upregulation in NSCLC tumor tissues compared with normal lung tissues using genome-wide miRNA expression profiling (Yanaihara et al., 2006). The 12 miRNAs could also be detected in plasma exosomes and showed similar signatures with tumor miRNAs, meaning the profile of plasma exosomal miRNAs parallels that of tumor-derived miRNAs. The diagnostic ability was not evaluated at that time. The prediction potential of these miRNAs was limited, as the ages and tumor stages of the enrolled patients were variable (Rabinowits et al., 2009). Remarkably, the miRNA levels inside the plasma exosomes of NSCLC patients were higher than those from healthy donors, which gave researchers confidence to carry out further studies (Rabinowits et al., 2009). Similarly, Wu et al. also demonstrated that serum exosomal miRNA-96 was upregulated in lung cancer patients and correlated with cancer stage (Wu et al., 2017), based on analysis of data previously collected from a cancer tissue miRNA array (Ma et al., 2011).

**TABLE 1:** Blood EV-miRNAs in lung cancer.

Category	*Cancer* type	miRNAs	Samples	EVs isolation methods	miRNA detection methods	Clinical potential	References
NSCLC	Lung adenocarcinoma of stage I-IV	hsamiR-17–3p, hsa-mir-21, hsa-mir-106a, hsa-mir-146, hsa-miR155, hsa-mir-191, hsa-mir-192, hsa-mir-203, hsa-mir-205	Plasma epithelial-derived exosomes and tumor tissue	Size exclusion chromatography and magnetic activated cell sorting using anti–epithelial cell adhesion molecule (EpCAM)	Selected from tissue miRNA arrays	Detectable, but failed to show diagnostic ability	Rabinowits et al. (2009)
		Hsa-miR-210, hsa-mir-212, and hsa-mir-214					
NSCLC	NSCLC of stage I-IV	let7f, miR-20b, miR-30e-3p	Plasma EVs	Super-paramagnetic beads coated with epithelial antibody	Taqman^®^ low-density arrays (365 miRNAs)	Discriminating and prognostic value	Silva et al. (2011)
NSCLC	Nodule including lung adenocarcinomas and lung granulomas of stage I	miR-378a, miR-379, mir-139–5p and miR-200b-5p	Plasma exosomes	ExoQuick exosome precipitation solution	Wide range microRNAs analysis (742 microRNAs)	Screen value	Cazzoli et al. (2013)
	Lung adenocarcinomas and lung granulomas of stage I	miR-151a-5p, miR-30a-3p, miR-200b-5p, miR-629, miR-100 and mir-154–3p	Plasma exosomes	ExoQuick exosome precipitation solution	Wide range microRNAs analysis (742 microRNAs)	Diagnostic value	Cazzoli et al. (2013)
NSCLC	NSCLC and non-NSCLC	Let-7b-5p, let-7e-5p, mir-24–5p, and mir-21–5p	Plasma exosomes	Ultracentrifugation	miRNA-seq using the NEBNext, multiplex small RNA library prep set for illumina (NEB)	Diagnostic value	Jin et al. (2017)
	Lung adenocarcinomas, SCC, and healthy volunteers	miR-181–5p, miR-30a-3p, miR-30e-3p, and mir-361–5p	Plasma exosomes	Ultracentrifugation	miRNA-seq using the NEBNext, multiplex small RNA library prep set for illumina (NEB)	Adenocarcinoma-specific	Jin et al. (2017)
	Lung adenocarcinomas, SCC, and healthy volunteers	miR-10b-5p, miR-15b-5p, and miR-320 b	Plasma exosomes	Ultracentrifugation	miRNA-seq using the NEBNext, multiplex small RNA library prep set for illumina (NEB)	SCC-specific	(Jin et al., 2017)
NSCLC	Lung adenocarcinomas	miR-21–5p, mir-126–3p, mir-140–5p	Serum exosomes	NA	RT-qPCR	Diagnostic value	Feng et al. (2018)
NSCLC and SCLC	NSCLC and healthy controls	Hsa-miR-451a, hsa-mir-486–5p, hsa-mir-363–3p, hsa-mir-660–5p, hsa-mir-15b-5p, hsa-mir-25–3p, hsa-mir-16-2-3p	Serum exosomes	NA	Small RNA sequencing kit of illumina	Diagnostic value	Poroyko et al. (2018)
	SCLC and healthy controls	Hsa-miR-1180	Serum exosomes	NA	Small RNA sequencing kit of illumina	Diagnostic value	Poroyko et al. (2018)
	SCLC and NSCLC	Hsa-miR-331–5p, hsa-mir-451a, hsa-mir-363–3p	Serum exosomes	NA	Small RNA sequencing kit of illumina	Diagnostic value	Poroyko et al. (2018)
NSCLC	NSCLC and healthy controls	Hsa-miR-660–5p	Blood exosomes	Ultracentrifugation	RT-qPCR	Diagnostic value	Qi et al. (2019)
NSCLC	Lung adenocarcinomas and healthy controls	38 miRNAs were significantly upregulated, e.g., mir-96–5p, miR-151a-5p, mir-342–3p, miR-374a-5p, mir-126–5p, and mir-16-2-3p; 37 miRNAs downregulated	Plasma exosomes	Ultracentrifugation	Illumina HiSeq3000 platform	Diagnostic value	Xue et al. (2020)
	Lung adenocarcinomas and healthy controls	miR-151a-5p, miR-10b-5p, mir-192–5p, miR-106b-3p, and miR-484	Plasma exosomes	Ultracentrifugation	Illumina HiSeq3000 platform	Prognostic value	Xue et al. (2020)
NSCLC	SCC and healthy controls	miR-181a-5p, mir-21–5p, miR-106a-5p, and mir-93–5p	Plasma exosomes	ExoQuick^™^ of system biosciences	Exiqon miRNA qPCR panel (168 miRNAs)	Failed to show difference	Shan et al. (2018)
NSCLC	NSCLC, non-malignant respiratory diseases, and healthy volunteers	miR-1246	Serum exosomes	NA	RT-qPCR	Diagnostic and prognostic value	Huang and Qu (2020)
NSCLC	Lung adenocarcinomas and healthy volunteers	31 exosomal miRNAs were upregulated and 29 exosomal miRNAs were downregulated, e.g., miR-7977, mir-98–3p	Serum exosomes	Total exosome isolation kit from serum of invitrogen	Agilent human miRNA microarray of agilent technologies	Diagnostic value	Chen et al. (2020)
NSCLC	Lung adenocarcinomas pre-operation and healthy volunteers	159 miRNAs significantly dysregulated in LA-pre samples vs. healthy volunteers; 24 miRNAs significantly dysregulated in LA-pre- vs. post-operation; 12 miRNAs showed same trend. E.g. mir-342–5p, mir-574–5p and mir-222–3p	Plasma exosomes	Ultracentrifugation	Small RNA sequencing using NEBNext^®^ multiplex small RNA library prep set for illumina	Diagnostic value	Han et al. (2020)
NSCLC	Lung adenocarcinomas and healthy volunteers	miR-1290	Serum exosomes	Total exosome isolation kits of invitrogen	RT-qPCR	Diagnostic and prognostic value	Wu et al. (2020b)
NSCLC	Both LA and SCC, health controls	miR-17–5p	Serum exosomes	ExoQuick of SBI	RT-qPCR	Diagnostic value	Zhang et al. (2019)
NSCLC	NSCLC, lung benign lesion (LBL), and healthy controls	miR-146a-5p and mir-486–5p	Serum exosomes	Exosome extraction kit of umibio shanghai	RT-qPCR	Diagnostic value	Wu et al. (2020a)
NSCLC	Patients with or without recurrence after surgery	miR-21 and miR-4257	Plasma exosomes	Ultracentrifugation	miRNA microarray using 3D-Gene human miRNA oligo chips ver. 20 of toray industries	Prognostic value	Dejima et al. (2017)
NSCLC	Lung adenocarcinomas and healthy volunteers	miR-23b-3p, miR-10b-5p and mir-21–5p	Plasma exosomes	ExoQuick exosome precipitation solution kit of SBI	qPCR	Prognostic value	Liu et al. (2017)
NSCLC	Platinum-resistant patients vs. platinum-sensitive patients	miR-425–3p	Serum exosomes	ExoQuick of SBI	RNA sequencing using NEBNext small RNA library prep set	Prognostic value	Yuwen et al. (2019)
NSCLC	Advanced EGFR/ALK wild-type (WT) NSCLC who received PD-1/pd-l1 inhibitors	Hsa-miR-320 days, hsa-mir-320c, hsa-mir-320b, hsa-mir-125b-5p	Serum exosomes	Ultracentrifugation	Small RNA next-generation sequencing	Prognostic value	(2020)

Subsequently, researchers increased their efforts to investigate the diagnostic and/or prognostic potential of EV-miRNAs in lung cancer (Table 1). One of the most impressive pieces of evidence was from a paper published in *ERJ* in 2011, where Garcia et al. analyzed the total EVs from the plasma of 28 matched NSCLC patients and 20 controls (Silva et al., 2011). Using miRNA arrays, they found that 10 miRNAs to be significantly differentially expressed, namely hsa-let-7 days, hsa-miR-223, hsa-miR-383, hsa-miR-192, hsa-miR-30e-3p, hsa-miR-301, hsa-let-7f, hsa-miR-572, hsa-miR-20b, and hsa-miR-345. Five of these (let-7f, miR-20b, miR-30e-3p, miR-223, and miR301) were randomly selected for further clinical evaluation on a new set of 78 NSCLC patients and 48 controls. Only let-7f, miR-20b, and miR-30e-3p were significantly decreased in the plasma EVs of NSCLC patients. Impressively, a higher level of let-7f and miR-30e-3p distinguished patients with resection (stages I, II, and IIIA) from those without (stages IIIB and IV). Meanwhile, researchers conducted long-term follow-up and found that higher plasma EV levels of miR-30e-3p and let-7f were associated with a higher rate of disease-free survival (DFS) and OS, respectively, (Silva et al., 2011). This evidence supports the important role of miRNA from plasma EVs as new biomarkers for lung cancer. Rodríguez also found that miR-223 presented a 200-fold increase in plasma exosomes derived from NSCLC compared to healthy controls (Rodriguez et al., 2014). Dr Cazzoli et al. attempted to evaluate a screening value for exosomal miRNA based on people with and without lung nodules, and in diagnosis, a value that might distinguish lung adenocarcinomas (LA) from benign lung granuloma (Cazzoli et al., 2013). Out of the 742 miRNAs assayed, miR-378a, miR-379, miR-139–5p, and miR-200b-5p showed a screening value with 97.5% sensitivity, 72.0% specificity, and an area under the curve (AUC) of 90.8%. Values determined for miR-151a-5p, miR-30a-3p, miR-200b-5p, miR-629, miR-100, and miR-154–3p were found to be distinct between LA and benign lung granuloma after the screening test. Meanwhile, a variety of studies have examined EVs’ diagnostic value for NSCLC. For example, Jin and Xie attempted to identify exosomal biomarkers that could discriminate between LA and SCC using a small RNA library system (Jin et al., 2017). The profiles of let-7b-5p, let-7e-5p, miR-24–5p, and miR-21–5p in plasma exosomes could distinguish NSCLC patients from non-NSCLC patients, with a sensitivity of 80.25%, specificity of 92.31%, and AUC value of 0.899 (Jin et al., 2017). Consistently, Feng isolated serum exosomes of LA patients and found that the AUC value for miR-21–5p was as high as 0.97 (Feng et al., 2018). miR-181–5p, miR-30a-3p, miR-30e-3p, and miR-361–5p were LA-specific overall, while miR-10b-5p, miR-15b-5p, and miR-320 b were SCC-specific (Jin et al., 2017). When establishing the diagnostic value based on a combination of miRNAs, the combination of miR-181–5p and miR-361–5p raised the AUC value to 0.936, with a sensitivity of 80.65% and a specificity of 91.67% in distinguishing LA patients from NSCLC patients (Jin et al., 2017). A similarly high diagnostic value of combined miR-320 b and miR-10b-5p was found to distinguish SCC patients from NSCLC patients (Jin et al., 2017). Another study regarding small cell lung cancer, NSCLC, and healthy controls found that hsa-miR-451a, hsa-miR-486–5p, hsa-miR-363–3p, hsa-miR-660–5p, hsa-miR-15b-5p, hsa-miR-25–3p, and hsa-miR-16-2-3p can distinguish NSCLC and control samples with high prediction accuracy using serum exosomes (Poroyko et al., 2018). Yongjian also confirmed that hsa-miR-660–5p was excessively released in plasma and exosomes in a total of 40 NSCLC patients (Qi et al., 2019). Meanwhile, hsa-miR-1180 can distinguish SCLC from controls. This is a remarkable finding, since there is no predictive molecule for treatment selection available so far (Fruh et al., 2013). Serum exosomal-miR-1180 might be an onco-molecule in SCLC. Notably, hsa-miR-331–5p, hsa-miR-451a, and hsa-miR-363–3p demonstrated excellent performance in discriminating between SCLC and NSCLC patients, with 100% sensitivity and specificity (Poroyko et al., 2018). To date, the diagnosis of lung cancer and follow-up of lung nodules have all relied on lung computed tomographic (CT) screening (National Lung Screening Trial Research et al., 2011). On the one hand, there is no doubt that CT scanning has reduced the mortality rate of lung cancer in recent decades compared to X-ray. On the other hand, whether the use of CT has been unnecessarily popularized is still a matter of debate, with 96.4% of the positive results determined using low-dose CT shown to be false positives after three rounds of screening. As several miRNAs have demonstrated perfect sensitivity and specificity in diagnosis, we strongly believe that EV-miRNAs could offer us a new strategy for lung cancer screening.

Several new studies on LA have recently been published. A recent study using exosomal miRNA profiling showed that 38 miRNAs were significantly upregulated in plasma exosomes of LA compared with a healthy control, e.g., miR-96–5p (about 800-fold), miR-151a-5p, miR-342–3p, miR-374a-5p, miR-126–5p, miR-16-2-3p, and miR-106b-3p (Xue et al., 2020), whereas 37 downregulated miRNAs were detected. miR-129–5p and miR-9-3p were highly enriched in the exosomes of the healthy control but undetectable in the plasma exosomes of LA patients. Sun et al. also demonstrated that blood exosomal miR-106 b was upregulated and associated with cancer stages and lymph node metastasis in lung cancer patients (Sun et al., 2020). Serum exosomal miR-1246 was also shown to have an early diagnostic value in distinguishing NSCLC from healthy controls with an AUC value of 0.827, and an AUC value of 0.757 when discriminating between NSCLC and non-malignant respiratory diseases (Huang and Qu, 2020). miR-7977 was confirmed by Chen et al. to be the miRNA with the highest fold change for upregulation in the serum exosomes of LA patients based on miRNA arrays (Chen et al., 2020). The diagnostic test showed that the AUC value of miR-7977 was 0.787 (Chen et al., 2020). miR-98–3p was demonstrated to be the most dramatic downregulated miRNA, with an AUC value of 0.719 (Chen et al., 2020). Another study regarding LA recruited three groups of patients comprising LA pre- and post-operation and healthy volunteers (Han et al., 2020). miRNA sequencing was performed using plasma exosomes representing all three groups. In total, 159 miRNAs were found to be significantly dysregulated in LA-pre samples vs. healthy volunteers; 24 miRNAs were significantly dysregulated in LA-pre- vs. post-operation; and a further 12 miRNAs showed the same trend, e.g., miR-342–5p (consistent with above study (Xue et al., 2020), miR-574–5p, and miR-222–3p (Han et al., 2020). The AUC values of miR-342–5p and miR-574–5p were both greater than 0.7 (Han et al., 2020). Wu et al. selected six miRNA candidates based on published papers that they assayed in the serum exosomes of 20 L A patients, including miR-21, miR-221–3p, miR-222–3p, miR-223, miR-638, and miR-1290 (Wu et al., 2020b). They found, however, that only miR-1290 showed significant differences in the exosomes of LA and healthy patients. ROC analysis using 90 patients demonstrated that exosomal miR-1290 showed better diagnostic efficiency than CEA, CYFRA21–1, and NSE in discriminating between LA patients and healthy controls (Wu et al., 2020b). Serum exosomal miR-146a-5p and miR-486–5p were also shown to have a diagnostic marker with an AUC value of 0.813 (sensitivity of 68.75%, specificity of 90%) and 0.886 (sensitivity of 70.83%, specificity of 95%), respectively, (Wu et al., 2020a).

For SCC, another study enrolled 102 SCC patients and 101 healthy controls (Shan et al., 2018). They performed miRNA microarray experiments using the plasma of 30 SCC and 10 healthy controls selected randomly. miR-181a-5p, miR-21–5p, miR-106a-5p, and miR-93–5p showed consistent upregulation and had overall AUC values of more than 0.7, which indicated their potential as diagnostic markers of SCC (Shan et al., 2018). However, the expression of these four miRNAs failed to show significance differences when examining plasma exosomes. Further research similar to Jin and Xie’s clinical study (Jin et al., 2017) on SCC will be needed to elucidate the physiological significance of EV-miRNAs. Apparently, one of our goal is to find new EV-miRNAs markers that contribute to early diagnosis. Therefore, clinical studies evaluating early stage (stage I and II) lung cancer are important. In the meantime, the specific EV-miRNAs that have diagnostic value in advanced stage lung cancer should be taken into prospective studies.

### Prognostic Value

Besides diagnostic value, EV-miRNAs are also potential prognostic markers. A miRNA microarray from NSCLC patients with or without recurrence after surgery revealed differences in the levels of several miRNAs (Dejima et al., 2017). Among these, patients with high plasma exosomal miR-21 and miR-4257 showed a significantly worse survival rate (Dejima et al., 2017). Plasma exosomal miR-23b-3p, miR-10b-5p, and miR-21–5p were independently associated with poor overall survival (Liu et al., 2017). Kaplan–Meier survival analysis showed that higher expression of miR-151a-5p, miR-10b-5p, miR-192–5p, miR-106b-3p, and miR-484 was associated with a lower survival rate (Xue et al., 2020), implicating them as prognostic biomarker candidates. Survival analysis showed that NSCLC patients with high serum exosomal miR-1246 had shorter OS and DFS than patients with low serum exosomal miR-1246 (Huang and Qu, 2020). Aside from diagnostic value, miR-1290 also demonstrated potential as a prognostic biomarker (Wu et al., 2020b). People with elevated exosomal miR-1290 had shorter progression-free survival (PFS) than those with lower levels (Wu et al., 2020b). High serum exosomal miR-378 was found to be indicative of shorter OS in NSCLC patients (Zhang and Xu, 2020). More cross-sectional studies and prospective studies should be conducted to evaluate the prognostic value of EV-miRNAs, such as monitoring the change of specific or a cluster of miRNAs and the relationship with DFS, OS, or PFS. The intervention of these EV-miRNAs could extend life for patients in the future.

### Treatment Responses

The use of EV-miRNAs has been examined for determining not only outcomes of lung cancer patients but also treatment responses. Yuwen et al. found that high miR-425–3p expression in circulating exosomes indicated disease progression and low responsiveness to platinum-based chemotherapy (Yuwen et al., 2019). A mechanistic study showed that miR-425–3p could target AKT1 (Yuwen et al., 2019). In recent years, the use of PD-1/PD-L1 inhibitors in immunotherapy has emerged. A total of 30 patients with advanced EGFR/ALK wild-type (WT) NSCLC who received PD-1/PD-L1 inhibitors were recruited by Peng et al., and plasma exosomal miRNAs were profiled using small RNA next-generation sequencing (Peng et al., 2020). A total of 155 differential expressed miRNAs were identified. Among these, a high level of hsa-miR-320 days, hsa-miR-320c, and hsa-miR-320 b appeared to be correlated with an unfavorable response to anti-PD-1 treatment (Peng et al., 2020). hsa-miR-125b-5p might be used as a potential indicator to monitor the efficacy of anti-PD-1 treatment (Peng et al., 2020). Numerous studies have investigated the targets or pathways related to differentially expressed miRNAs in lung cancer (Poroyko et al., 2018; Xue et al., 2020). One study identified the biological processes of serum exosomal miRNAs in both NSCLC and SCLC patients (Poroyko et al., 2018). As expected, the top biological functions of exosomal miRNAs were found to be related to cancer and organ injury in NSCLC, SCLC, or SCLC after chemotherapy treatment. As for molecular and cellular functions, the most dramatic example was of cellular assembly and organization in SCLC samples, which did not even show up in the top five functions for NSCLC samples. The exosomal miRNA-related cellular functions may contribute to the rapid progress and early metastasis of SCLC. After chemotherapy treatment, the analysis of cellular growth and proliferation functions showed a switch to the top differential cellular functions in SCLC patients, which matched the good response to chemotherapy in SCLC patients (Poroyko et al., 2018). This gives us confidence in evaluating the top exosomal-miRNA-related pathways for developing promising targeted drugs, such as molecular mechanisms of cancer, glioblastoma multiforme signaling, glioblastoma signaling, PI3K/AKT signaling, and glucocorticoid receptor signaling (Poroyko et al., 2018). Nonetheless, aberrancies in more well-recognized molecules, such as EGFR mutation and ALK rearrangement in NSCLC, may be involved in lung cancer drug resistance and should be further studied, along with targeted therapy. Almost all patients who had EGFR mutation and been treated with EGFR-TKIs led to resistance (Planchard et al., 2018). Closer analysis of T790 M mutation is required as it accounts for over 50% of first and second generation EGFR-TKIs resistances (Planchard et al., 2018). Notably, one study suggested that exosomal-miR-522–3p may induce first-generation EGFR-TKI resistance in NSCLC cells (Liu et al., 2020c). In the current guideline, cell-free DNA blood test for detecting T790 M mutation is a common way, but has a low sensitivity. Thus, in many cases, a second biopsy with lung tissue was needed be performed (Planchard et al., 2018). However, the second biopsy is hard to be accomplished, especially for those patients with severe progression and high performance status score. In this context, EV-miRNAs can be a promising marker that evaluate drug resistance efficiently. This will improve our scientific understanding for approaching EV-based lung cancer precision therapy.

### Combination for Higher Diagnostic Value

Doing a diagnostic test based only on a single miRNA may not result in a satisfied AUC being reached. Combining different items, however, may help to improve the diagnostic value (Table 2). As shown before, combined miR-181–5p and miR-361–5p raised the AUC value to 0.936, with a sensitivity of 80.65% and a specificity of 91.67% in identifying LA patients from NSCLC patients (Jin et al., 2017). Meanwhile, the combination of miR-320 b and miR-10b-5p could be used to establish a diagnostic value for distinguishing SCC patients from NSCLC patients (Jin et al., 2017). Zhang et al. also found that the combination of miR-17–5p and three tumor markers (CEA, CYFRA21–1, and SCCA) could increase AUC from 0.738 to 0.86 (Zhang et al., 2019). A similar conclusion was reached for serum exosomal miR-216 b (Liu et al., 2020b). Although serum exosomal miR-146a-5p and miR-486–5p were good potential markers with high specificity, the combination of four serum miRNAs (miR-21–5p, miR-141–3p, miR-222–3p, and miR-486–5p) and these two exosomal miRNAs increased the AUC to 0.96 (Wu et al., 2020a). Similarly, other evidence showed that regardless of the specific combination of serum exosomal miR-378 and CEA or multiple miRNAs, an improvement in the diagnostic value of NSCLC patients can be achieved (Chen et al., 2020; Zhang and Xu, 2020). Nevertheless, extensive clinical research should be performed to investigate and identify convincing miRNAs that can be combined for the diagnosis of lung cancer. Additionally, development of a fast detection kit for assaying the combined miRNAs would be conducive to clinical application.

**TABLE 2:** Combination for higher diagnostic value in blood.

miRNAs	Clinical potential	Samples	Diagnostic value	References
Combination of miR-181–5p and miR-361–5p	Identify LA patients out of NSCLC patients	Plasma exosomes	AUC value was 0.936, with a sensitivity of 80.65% and a specificity of 91.67%	Jin et al. (2017)
Combination of miR-320 b and miR-10b-5p	Distinguish SCC patients out of NSCLC patients	Plasma exosomes	AUC value was 0.911, with a sensitivity of 83.33% and a specificity of 90.32%	Jin et al. (2017)
Combination of mir-17–5p and three tumor markers (CEA, CYFRA21–1 and SCCA)	Diagnosis of NSCLC	Serum exosomes	AUC value was 0.86	Zhang et al. (2019)
Combined detection of 4 serum miRNAs (mir-21–5p, mir-141–3p, mir-222–3p, mir-486–5p) with serum exosomal miR-146a-5p and mir-486–5p	Diagnosis of NSCLC	Serum exosomes	AUC value was 0.96	Wu et al. (2020a)
Combination of exosomal miR-7977 and miR-98–3p	Diagnosis of LA	Serum exosomes	AUC value was 0.816	Chen et al. (2020)
Combination of serum exosomal miR-378 expression and carcinoembryonic antigen (CEA)	Diagnosis of NSCLC	Serum exosomes	AUC value was 0.886	Zhang and Xu (2020)

## Lung Specific Liquids: BALF and Pleural Lavage

The use of BALF represents a direct approach to assessing the lungs which is essential in the diagnosis of lung infection, lung cancer, and immunological lung diseases. The role of BALF EVs in the diagnosis of lung cancer is still unclear. Total BALF EVs were isolated in smokers and lung cancer by Wu et al. (Wu et al., 2019). Lung cancer patients were shown to have a higher level of BALF EVs, also indicating a greater amount of cargo that is held within the EVs (Wu et al., 2019). miRNA profiles showed dysregulation in both smokers vs. controls and lung cancer patients vs. smokers (e.g., miR-107, miR-126, miR-124–3p) (Wu et al., 2019). Rodríguez et al. collected exosomes of both plasma and BALF from NSCLC patients, and miRNA profiling was performed (Rodriguez et al., 2014). miR-7-5p, miR-424–5p, miR-196b-5p, miR-124–3p, miR-185–5p, and miR-18a-5p were the most significantly upregulated (fold change >5) miRNAs in tumor BALF (Rodriguez et al., 2014). Kim et al. detected several miRNAs in the BALF exosomes of LA patients and found that miR-126 and Let-7a levels were significantly higher in the LA patients than in the control subjects (Kim et al., 2018). All the above papers regarding BALF EV-miRNA did not include a diagnostic test. Further studies should be conducted on BALF EV-related biomarkers.

Pleural lavage is one of the symptoms of lung cancer or lung infection. LA-related malignant pleural effusion (MPE) is a commonly the initial reason to visit the hospital and is relatively easily contracted. In 2019, Canal et al. conducted pleural lavage of lung cancer patients and controls (Roman-Canal et al., 2019). EVs were isolated and miRNA profiling was performed. In total, 14 miRNAs were found to be dysregulated, including miR-150–5p, miR-144–5p, miR-1-3p, and miR-584–5p (Roman-Canal et al., 2019). miR-150–5p, miR-144–5p, and miR-1-3p were evaluated as being diagnostic EV-miRNA markers for lung cancer (Roman-Canal et al., 2019). Tamiya et al. isolated exosomes from LA-related MPE and benign (non-neoplastic) pleural effusion (BPE) (Tamiya et al., 2018). The expression of exosomal miR-182 and miR-210 was significantly higher in the LA-MPE than in the BPE samples. It is worth noting that the diagnostic performance of miR-182 was good, with an AUC of 0.87, sensitivity of 92.7%, and specificity of 73.3% (Tamiya et al., 2018). miRNA profiling of LA-MPE and BPE showed 17 exosomal miRNAs to be differentially expressed (Hydbring et al., 2018). The top miRNAs displayed an average AUC of 0.95, such as miR-375, miR-200b, miR-200c, and miR-141 (Hydbring et al., 2018) (Table 3). Although no result has been posted yet, the phase two clinical trial (NCT03228277) is recruiting T790 M-positive confirmed in BALF samples of NSCLC patients. This can be great supporting evidence that BALF and pleural lavage are promising liquids for lung cancer patients.

**TABLE 3:** Lung specific liquids: BALF and pleural lavage.

Category	Cancer type	miRNAs	Samples	EVs isolation methods	miRNA	Clinical potential	References
NSCLC	NSCLC, smokers, and healthy controls	31 miRNAs dyregulation, e.g., miR-107, miR-126, mir-124–3p	BALF EVs	Ultracentrifugation	Agilent bioanalyzer RNA 6000 chip	NA	Wu et al. (2019)
NSCLC	NSCLC patients and patients with nontumor pathology	miR-7-5p, mir-424–5p, miR-196b-5p, mir-124–3p, mir-185–5p, miR-18a-5p	BALF exosomes	Ultracentrifugation	miRNA profiles	NA	Rodriguez et al. (2014)
NSCLC	LA and controls with non-tumor pathology	miR-126 and Let-7a	BALF exosomes	ExoQuick exosome precipitation solution of SBI	qPCR	NA	Kim et al. (2018)
NSCLC	NSCLC and control with BPE	14 miRNAs were found dysregulated including mir-150–5p, mir-144–5p, mir-1-3p, mir-584–5p	Pleural lavage EVs	Ultracentrifugation	Sanger mirbase v14 of 754 miRNAs	Diagnostic value	Roman-Canal et al. (2019)
NSCLC	LA related MPE and BPE	miR-182 and miR-210	Pleural lavage exosomes	Total exosome isolation reagent of invitrogen	qPCR	Diagnostic value	Tamiya et al. (2018)
NSCLC	LA related MPE and BPE	17 differential expression of exosomal miRNAs, e.g., miR-375, miR-200b, miR-200c, miR-141	Pleural lavage exosomes	NA	miRNA array	Diagnostic value	Hydbring et al. (2018)

## Methods and Questions

EV-related miRNAs have been a topic of focus as a liquid biopsy tool. Some papers on standard and emerging were recently published. Giallombard et al. presented a protocol for the used of exosomes in diagnosis in 2016, which included exosome isolation from plasma, exosome characterization, exosomal RNA extraction and quantification, reverse transcription, and real-time PCR (Giallombardo et al., 2016). They suggest using mir-1228–3p as an endogenous control. However, it might be further evaluated and demonstrated as a general control in various physiological conditions. We had published a review paper regarding different methods to isolate EVs from BALF (Carnino et al., 2019). So far, miRNA profiling, sequencing, and arrays have been extensively used in various EV-miRNA studies, and a relatively large number of samples were needed for analysis. A next-generation RNA sequencing with small RNA libraries is now a widely used technique. 1 μg of total RNA isolated from each EV sample can be applied for the small RNA sequencing using the Illumina MiSeq platform (Huang et al., 2013; Hannafon et al., 2016; Zhang et al., 2020). Ma et al. developed a new exosomal miRNA detection method based on surface-enhanced Raman scattering, which presents high sensitivity to blood samples (Ma et al., 2018). Researchers from the State University of New York developed another non-PCR-based detection system, which is a tethered cationic lipoplex nanoparticle (tCLN) biochip (Liu et al., 2020a). Higher sensitivity, specificity, and AUC than those of the traditional qPCR method were demonstrated (Liu et al., 2020a).

As shown in Table 1, there were few miRNAs that overlapped in the different studies. One possible reason is that the sample type and analysis methods were different in similar studies. A meta-analysis reviewed liquid exosomes in lung cancer patients of 13 articles and found that the diagnostic accuracy of serum samples was higher than that of plasma samples (Song et al., 2019). The other difference might be a technical method to isolate EVs from biofluids. Although meta evidence supported the fact that the commercial kit method had a better diagnostic value, this still needs to be elucidated. More studies using other methods should be included for better analysis. A consensus of sample types (e.g., plasma or serum) and EV-isolation methods should be made before introducing EV-miRNAs into clinical use. Furthermore, miRNA clusters should be evaluated for broad application in liquid biopsy for lung cancer instead of single miRNAs.

## Discussion and Prospective

There are limitations to the research of the EV-containing miRNAs. First, EVs are actually highly heterogeneous. Thus, it would be necessary to develop a method to isolate a particular population of EVs in the body fluids. For example, in our previous study, we developed a method to purify a miRNA-rich EV population from BALF (Lee et al., 2019). Second, it is extremely hard to isolate pure EVs without any contaminations. Until recently, there are many methodological trials to improve the purify of EVs (Carnino et al., 2019). Therefore, to clearly detect EV-miRNAs as a biomarker, various isolation methodologies should be tried in the further clinical studies. Cross-sectional studies focused on the EV-miRNAs using different isolation methods in lung cancer patients may give us chances to address these questions. Subsequently, standard technical guideline should be established for the clinical practice in the future.

With the emerging of next-generation sequencing (NGS), the sequencing technique had played an essential role in the screening of oncogenes, such as EGFR mutation, BRAF mutation, ALK and ROS1 rearrangements. This enables more efficient and multiplex oncogenic target screening, which results in reducing the wait time for patients. Nevertheless, the detection and monitoring of protein levels for those biomarkers are still required. Thus, profiling EV-miRNAs which is a RNA-based analysis can provide much better performance for the clinical practice. Notably, hsa-miR-320 cluster was predicted to reveal a poor response to PD-L1 inhibitors (Peng et al., 2020). Pembrolizumab was the first approved drug as a PD-L1 inhibitor. In the KEYNOTE-407 study, the combination of pembrolizumab plus chemotherapy was recommended as the first-line standard treatment in patients with metastatic SCC, regardless of PD-L1 expression (West et al., 2019). Thus, the TPS score of PD-L1 expression in each type of NSCLC may not fully reflect the patient status. EV-miRNA cluster detected using RNA sequencing can be a powerful next-generation biomarker for the response of immunotherapy, or epoch-making treatment target for lung cancer especially for SCLC.

As per Response Evaluation Criteria in Solid Tumors (RECIST) v1.1, RECIST can be used to evaluate responses of targeted therapies. Although this still is the standard method of clinical practice, it is still debatable (Planchard et al., 2018). As we talked above, the T790 M mutation is the most common cause of EGFR-TKI resistance. There already is a study that found a certain exosomal-miRNA, miR-522–3p, accounts for gefitinib resistance *in vitro* (Liu et al., 2020c). However, sufficient evidence should be explored and provided before we adapt EV-miRNA to clinical practice. Over the past decades, there has been an emerging trend in clinical trials regarding EVs or exosomes and their roles in lung cancer. Biomarkers for diagnosis, prognosis and treatment responses are needed. As we mentioned in the first part, several clinical trials had been registered online, trying to examine EV-miRNAs and their roles in lung cancer in several conditions (such as NCT03542253, NCT04529915, NCT04629079, NCT04427475). Regretfully, no advanced clinical trials were available now. All the clinical trials are still in the recruiting stage or pre-recruiting stage, which shows just how cutting-edge this topic is. Three clinical trials regarding exosomes or EVs in lung cancer are found to be in phase II (NCT01159288, NCT03228277, and NCT01629498). However, EV-miRNAs were not listed as outcome measures in these three advanced clinical trials. As the importance of EV-miRNAs in lung cancer is rising, they may run RNA sequencing of EVs as additional outcomes.

## Summary

In summary, EV-miRNA is now considered to be a promising biomarker for lung cancer based on the fact that EVs contain stable miRNAs and are actively released from stimulated cells in our body. Emerging evidence shows that EV-miRNAs have a high diagnostic and prognostic value whether they are in blood or BALF or obtained from pleural lavage of lung cancer patients. Although more studies are needed for establishing EV-miRNAs as a novel tool for clinical analysis, this review shows that EV-miRNA is a strong candidate for lung cancer diagnosis, prognosis, and treatment responses.

## Abbreviations

EVs, extracellular vesicles; miRNAs, microRNAs; BALF, bronchoalveolar lavage fluid; NSCLC, non-small cell lung cancer; SCC, squamous cell carcinoma; LA, ung adenocarcinomas; SCLC, small cell lung cancer; DFS, disease-free survival; OS, overall survival; CT, computed tomographic; AUC, area under the curve; MPE, malignant pleural effusion.
